# Decoding the temporal nature of brain GR activity in the NFκB signal transition leading to depressive-like behavior

**DOI:** 10.1038/s41380-021-01016-1

**Published:** 2021-01-22

**Authors:** Young-Min Han, Min Sun Kim, Juyeong Jo, Daiha Shin, Seung-Hae Kwon, Jong Bok SEO, Dongmin Kang, Byoung Dae Lee, Hoon Ryu, Eun Mi Hwang, Jae-Min Kim, Paresh D. Patel, David M. Lyons, Alan F. Schatzberg, Song Her

**Affiliations:** 1 Seoul Centre, Korea Basic Science Institute, Seoul, South Korea; 2 Department of Life Science, Ewha Womans University, Seoul, South Korea; 3 Department of Physiology, School of Medicine, Kyung Hee University, Seoul, South Korea; 4 Neuroscience Centre, Korea Institute of Science and Technology, Seoul, South Korea; 5 Center for Functional Connectomics, Korea Institute of Science and Technology, Seoul, South Korea; 6 Department of Psychiatry, Chonnam National University Medical School, Seoul, South Korea; 7 Department of Psychiatry, Molecular and Behavioral Neuroscience Institute, University of Michigan Medical Centre, Ann Arbor, MI USA; 8 Departments of Psychiatry, Stanford University Medical Centre, Stanford, CA USA

**Keywords:** Molecular biology, Biological techniques

## Abstract

The fine-tuning of neuroinflammation is crucial for brain homeostasis as well as its immune response. The transcription factor, nuclear factor-κ-B (NFκB) is a key inflammatory player that is antagonized via anti-inflammatory actions exerted by the glucocorticoid receptor (GR). However, technical limitations have restricted our understanding of how GR is involved in the dynamics of NFκB in vivo. In this study, we used an improved lentiviral-based reporter to elucidate the time course of NFκB and GR activities during behavioral changes from sickness to depression induced by a systemic lipopolysaccharide challenge. The trajectory of NFκB activity established a behavioral basis for the NFκB signal transition involved in three phases, sickness-early-phase, normal-middle-phase, and depressive-like-late-phase. The temporal shift in brain GR activity was differentially involved in the transition of NFκB signals during the normal and depressive-like phases. The middle-phase GR effectively inhibited NFκB in a glucocorticoid-dependent manner, but the late-phase GR had no inhibitory action. Furthermore, we revealed the cryptic role of basal GR activity in the early NFκB signal transition, as evidenced by the fact that blocking GR activity with RU486 led to early depressive-like episodes through the emergence of the brain NFκB activity. These results highlight the inhibitory action of GR on NFκB by the basal and activated hypothalamic-pituitary-adrenal (HPA)-axis during body-to-brain inflammatory spread, providing clues about molecular mechanisms underlying systemic inflammation caused by such as COVID-19 infection, leading to depression.

## Introduction

A growing body of evidence suggests that inflammation has a pathophysiological role in depression. During inflammatory processes, nuclear factor-κ-B (NFκB) plays a major role in cellular responses to a wide variety of stimuli. As a counterpart, when glucocorticoid receptors (GRs) bind glucocorticoids (GCs), they exert essential immunosuppressive and anti-inflammatory actions. Therefore, it has been postulated that dysfunctional GR activity causes depression, due to crosstalk problems between NFκB and GR, as shown in our previous reports on dampened GR activity [1]. In the study on their targeted transcripts, however, the exact brain mechanisms of this crosstalk have remained unresolved by jumbled gene expressions caused by different structures of their gene promoters [2, 3]. Individual variation particularly induced by endpoint analysis is another difficult barrier for crosstalk readings [4, 5]. Therefore, rather than attempting to investigate transcriptional NFκB-GR, we focused on the longitudinal monitoring of the signal NFκB-GR interplay using in vivo imaging techniques.

Although transgenic reporter mice are an alternative solution to the direct monitoring of biological processes [6, 7], in vivo real-time monitoring of their activation has been inaccessible, partly due to the difficulty in spatial resolution, that could describe such events. Their clonal ubiquity leads to poor special resolution and severely impedes pinpoint monitoring of transcriptional activity. Particularly, spacious bioluminescence is a major disadvantage in studying anatomical brain function in which those functions can be attributed to different regions of the brain or even the same cell type [8, 9]. Other strategies, such as Cre-Lox recombination [10] and the non-viral gene delivery system [11], also have unresolved problems with bioluminescence. Herein, using pinpoint stereotaxic injection with improved lentivirus-based luciferase (Luc) reporters, we identify the pinpoint dynamic activities of GR and NFκB, allowing precise signal trajectories and timing of NFκB-GR interplay.

We targeted the infralimbic prefrontal cortex (IL-PFC, corresponding to the ventromedial PFC in humans) as a representative anatomical brain region associated with depression [12, 13]. The dermis was targeted as a representative body region in which various aspects of the immune system actively maintains skin homeostasis [13, 14]. We used a lipopolysaccharide (LPS)-induced depressive mouse model that shows time-dependent behaviors such as early symptoms of sickness and late transient depressive-like behavior on a short timescale [15]. Understanding the NFκB-GR interplay during the behavioral outcomes helps to decipher the molecular mechanisms underlying the etiology of inflammation-associated depression.

## Method summary

To monitor signal activity with the IVIS 200 imaging system (PerkinElmer Company, Alameda, USA), ICR mice were injected with one of the lentiviral reporters (NFκB-Luc2CP, GR-Luc2CP, or elongation factor (EF)1α-Luc2CP, Supplementary Fig. 1). Meanwhile, the cellular location of the signal was determined through immunohistochemical staining in which NFκB and GR signal activities were mainly detected in NeuN^+^ neuronal cells in the IL-PFC (Supplementary Fig. 2). [1, 16]. In the monitoring of NFκB and GR activities, three bioluminescence experiments were performed to investigate the LPS effect, the LPS effect on the RU486, or adrenalectomy (Supplementary Fig. 3b–d). The average signal obtained from different animals injected with the EF1α-Luc reporter was used as the reference for the normalization of two signals. The BLI data from an individual mouse, combined from three independent studies were analyzed by one- or two-way analysis of variance (ANOVA) tests, and the statistical significance between groups was determined by Sidak’s multiple comparison test; otherwise, Student’s *t*-test was used. Detailed materials and methods and associated references are provided in the Supplementary Information.

## Results

### Distinguishing NFκB activation in the IL-PFC and dermis by an improved NFκB-reporter

**Fig. 1:**
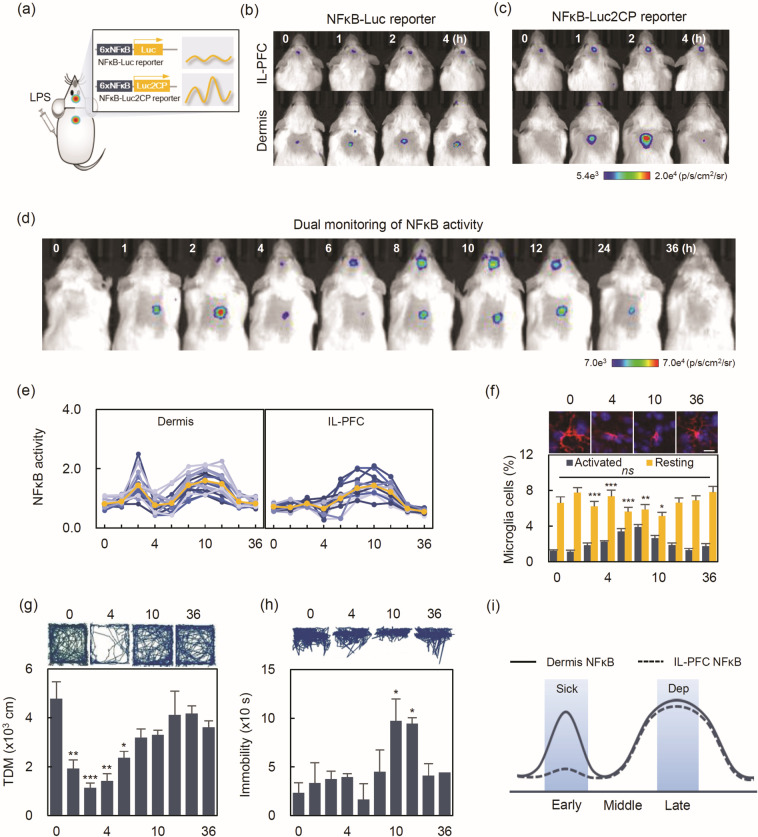
Different NFκB responses in the IL-PFC and dermis. **a** Schematic illustrating an improved NFκB reporter for the monitoring of dynamic transcriptional activity in a living mouse. Comparative bioluminescent images (BLIs) of (**b**) NFκB-Luc or (**c**) NFκB-Luc2CP reporter. NFκB activity was monitored by single-monitoring in the IL-PFC or dermis after intraperitoneal injection of 1 mg/kg LPS. **d** Representative BLIs of NFκB dual-monitoring by the NFκB-Luc2CP reporter. **e** Individual NFκB profiles of the dermis (left panel) and IL-PFC (right panel). The orange line represents the average NFκB activity at each time point (*n* = 13). **f** Representative fluorescence images of microglia (top, scale bar = 10 μm) and quantification of activated and resting microglia (bottom) in the IL-PFC. Immunohistochemical staining with Iba1 and DAPI. Time course of behaviors for (**g**) total distance moved in the OFT and (**h**) immobility time in the FST with representative tracking paths. Data are represented as the mean ± standard error of measurement [*n* = 8/group, except for 6 h post-LPS in the OFT (*n* = 7/group) and 36 h post-LPS in the FST (*n* = 7/group)]. **i** The trajectories of the two NFκB activities. Statistical significance was determined by Student’s *t*-test (*P < 0.05, **P < 0.01, ***P < 0.001, ns = non-significant vs control). IL-PFC infralimbic prefrontal cortex, Sick sickness behavior, Dep depressive-like behavior, FST forced swimming test, OFT open field test, TDM total distance moved.

To monitor NFκB activity in vivo, first, we modified the previous lentiviral-based GR-Luc reporter by replacing 5×GRE-AIEP with a 6×NFκB-TATA DNA fragment, designated NFκB-Luc reporter (Supplementary Fig. 1a). During the monitoring of NFκB activity by bioluminescent signal image (BLI), however, we noticed that the reporter was insufficient for monitoring NFκB activation in the IL-PFC or the dermis (Fig. 1a, b). The temporal resolution of the NFκB-Luc reporter, associated with the long half-life of luciferase [17], hindered the accurate monitoring of NFκB activation. Thus, we developed an improved NFκB-Luc2CP reporter by replacing Luc with Luc2CP as in the previously reported GRE-Luc2CP [16], expecting high temporal resolution with increased intensity. The monitoring of NFκB activity with this improved reporter showed clear dermal NFκB activation at 2 h post-LPS; however, there was still no NFκB activation observed in the IL-PFC (Fig. 1c), which may be an in vivo evidence for different responses of NFκB in the IL-PFC and dermis. This difference was confirmed by a qPCR assay demonstrating decreased expression of NFκB-responsive genes (interleukin-1β, granulocyte-macrophage colony-stimulating factor; GM-CSF, and tumor necrosis factor; TNF α) (Supplementary Fig. 4a, b). The lower NFκB response in the IL-PFC compared to the dermis was also confirmed, as evidenced by the left-shifted distribution of the 32 NFκB-response gene expression (Supplementary M & M and Supplementary Fig. 4c).

### Tissue-specific NFκB activation in behavioral outcomes

To exclude possible errors from intra-individual variations, an NFκB dual-monitoring assay was performed on the IL-PFC and dermis of the same subject up to 36 h post-LPS. As shown by the BLIs (Fig. 1d) and NFκB profiles (Fig. 1e), differential NFκB activation was verified. Two hours post-LPS, dermal NFκB activity showed a clear peak, but no activity was shown in the IL-PFC. Two-way ANOVA with repeated measures (ANOVA RM) showed significant effects of tissue (*F*_1,24_ = 6.04, *P* < 0.05), time (*F*_9, 216_ = 22.28, *P* < 0.001), and tissue × time interaction (*F*_9,216_ = 5.52, *P* < 0.001). Sidak multiple comparison tests revealed that the NFκB activity in the IL-PFC was significantly lower than in the dermis at 2 h post-LPS (*P* < 0.001, mean difference = 0.69). Meanwhile, during 8–12 h post-LPS, NFκB was equally activated in both the IL-PFC and dermis without significant difference (*P* > 0.05 at 8, 10, and 12 h). Immunohistochemical (IHC) analysis with IL-PFC tissues showed that activated microglial cells were also significantly increased during the late-phase (Fig. 1f).

Open field test (OFT) and forced swimming (FST) were performed at the indicated time points to elucidate sickness and depressive-like behavioral links to signal activities, respectively. Consistent with previous findings [18–20], the LPS challenge induced time-dependent behavioral outcomes, as shown an increase in OFT total distance moved (TDM) that occurred over 1–6 h post-LPS (Fig. 1g) and a decrease in FST immobility over 10–12 h post-LPS challenge (Fig. 1h). The time-dependent behavioral outcomes appeared to be associated with NFκB activation in a tissue-specific manner. A comparative trajectory showed that dermal NFκB activation correlated with the early-phase where increased TDM was observed. In contrast, IL-PFC NFκB activation correlated with the late-phase where decreased immobility was observed (Fig. 1i).

### Two faces of GR in the inhibition of NFκB activity

**Fig. 2:**
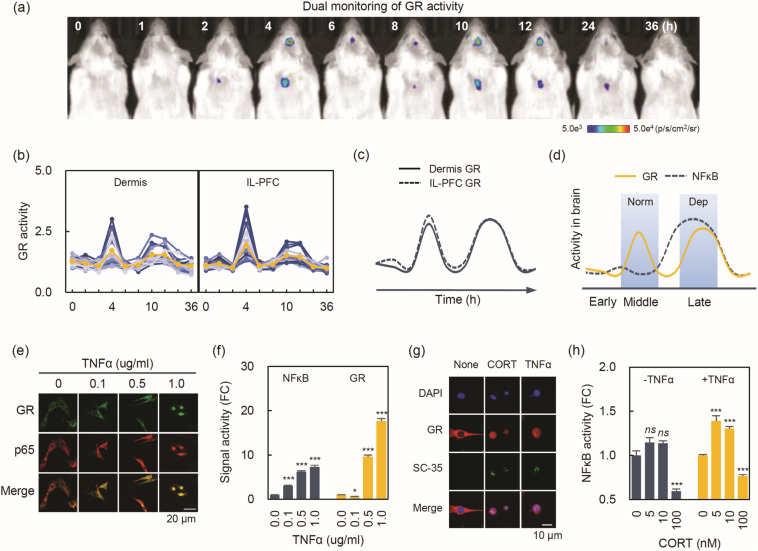
Distinct GR involvement in the inhibition of NFκB activity. **a** Representative bioluminescent images (BLIs) of GR dual monitoring by the GRE-Luc2CP reporter. **b** Individual GR profiles of the dermis (left panel) and IL-PFC (right panel). The orange line represents the average GR activity at each time point (*n* = 15). **c** The trajectories of the two GR activities, and (**d**) the trajectories of the NFκB and GR activities in the IL-PFC. **e** Representative confocal images of nuclear GR translocation by TNFα in H19-7 cells. The cells were stained for GR (green) and p65 (red). **f** The effect of TNFα on NFκB and GR activities. To evaluate the effects, H19-7 cells were infected with each Luc2CP reporter and then incubated for 36 h in the presence of the indicated doses of TNFα (*n* = 4/group for in vitro tests). **g** Representative confocal images of nuclear GR translocation and SC-35 as a nuclear speckle marker induced by CORT or TNFα treatment in H19-7 cells. The cells were stained for GR (red) and SC-35 (green). **h** The effects of CORT on NFκB activity. To evaluate the effects, H19-7 cells were infected with NFκB Luc2CP reporter and then incubated for 36 h in the presence or absence of the TNFα by the presence of the indicated doses of CORT (*n* = 4/group for in vitro test). Each signal activity was divided by an average of EF1α-Luc2CP signals. Statistical significance was determined by Student’s *t*-test (*P < 0.05, ***P < 0.001 vs control). IL-PFC infralimbic prefrontal cortex; CORT corticosterone. Norm normal behavior; Dep depressive-like behavior (color figure online).

In the trajectory of dermal NFκB, we noticed a discontinuance during the middle-phase (Fig. 1i); this is likely due to the inhibitory actions of GR on NFκB, as evidenced by a dramatic increase in plasma CORT at 2 h post-LPS (Supplementary Fig. 5a). To demonstrate this, we performed dual monitoring of GR activity with the GRE-Luc2CP reporter (Fig. 2a) [16]. Unlike the differences shown in NFκB activation (Fig. 1e, i), GR activation showed no difference between the IL-PFC and dermis across all time points (Fig. 2b, c; tissue × time interaction: *F*_9,252_ = 0.93, *P* = 0.50), but significant GR activations were observed (time: *F*_9, 252_ = 42.11, *P* < 0.001) during the middle and late-phase. As expected, the trajectory of GR was opposite to that of NFκB during the middle-phase, suggesting that the middle-phase GR activation plays a key role in modulating excessive inflammation upon binding to high levels of GC. In contrast, it seems that late-phase GR activity has no inhibitory action, as evidenced by similar trajectories of GR and NFκB and consequent depressive-like behavior (Fig. 2d).

The conflicting results of GR inhibitory action on NFκB were corroborated by in vitro experiments with H19-7 cells. TNFα treatment significantly increased GR and NFκB nuclear translocations and their corresponding activities (Fig. 2e, f), suggesting cytokine-induced GR activation. Interestingly, both CORT and TNFα induced SC35-positive speckles (Fig. 2g), implying involvement of transcriptional mechanism in the TNFα-driven gene regulation as well as CORT-driven gene regulation [21]. We also found that the activated NFκB induced by TNFα was eradicated by the high dose of CORT treatment (Fig. 2h). Accordingly, in in vivo experiments with systemic LPS challenge, the transient depressive-like behavior during the late-phase was eliminated by CORT pre-treatment (Supplementary Fig. 6), suggesting a restored inhibitory action by CORT [22–24].

### Systemic blockade of GR activity leading to activation of NFκB in the IL-PFC

**Fig. 3:**
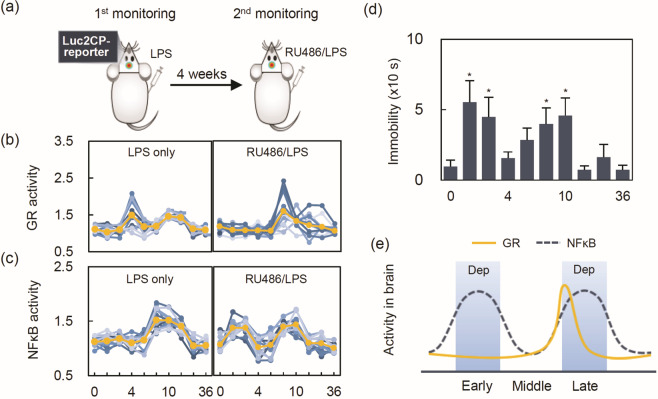
RU486 induced early brain NFκB activation and corresponding depressive-like behavior. **a** Schematic illustrating repeated analysis with single-monitoring in IL-PFC. **b** Individual GR profiles of 1 mg/kg LPS (left panel) and 10 mg/kg RU486 + 1 mg/kg LPS (right panel). **c** Individual NFκB profiles of 1 mg/kg LPS (left panel) and 10 mg/kg RU486 + 1 mg/kg LPS (right panel). The orange line represents the average GR activity at each time point (*n* = 12). Time course of behavior for (**d**) immobility time in the FST. Data are represented as the mean ± standard error of measurement [*n* = 8/group, except for 12 h post-LPS (*n* = 7/group)] (**e**) the trajectories of NFκB and GR activities in the LPS/RU486-treated mice. Statistical significance was determined by Student’s *t*-test (*P < 0.05 vs control). IL-PFC infralimbic prefrontal cortex, LPS lipopolysaccharide, FST forced swimming test, Dep depressive-like behavior (color figure online).

Basal GR activity was blocked by RU486 before LPS treatment, to determine if low GR activity is sufficient to inhibit NFκB activity in the IL-PFC (Fig. 3a). As expected, repeated single-monitoring assays showed that GR activity was significantly reduced in the RU486-treated group compared with the control group during the early-phase (Fig. 3b; time × treatment: *F*_9, 110_ = 6.78, *P* < 0.001, *P* < 0.001 at 4 and 8 h). Reversely, NFκB activity was significantly increased (Fig. 3c; time × treatment: *F*_9, 99_ = 8.34, *P* < 0.001; *P* < 0.05 at 1, 2, 4, and 12 h). Depressive-like behavior was also observed during the early and late-phases (Fig. 3d, e).

The rebound of GR activation after RU486 clearance seemed to be induced by cytokines, as shown in the late-phase GR activity by LPS (Fig. 3b) because depressive-like behaviors were measured during this phase. In line with this, the rebound in GR activation was also observed in the RU486 experiments in ADX mice (Supplementary Fig. 7). In the test of a direct GR involvement in the IL-PFC using RU486 brain infusion, similar results of depressive-like behavior were obtained without a dramatic increase in plasma CORT compared to the LPS-treated group (Supplementary Fig. 8). Meanwhile, there were dramatic CORT increases in RU486/LPS *i.p.* injection, irrespective of the phase (Supplementary Fig. 5b), as reported by previous studies [25–28].

### Transcriptional links to NFκB-GR signal interplay

**Fig. 4:**
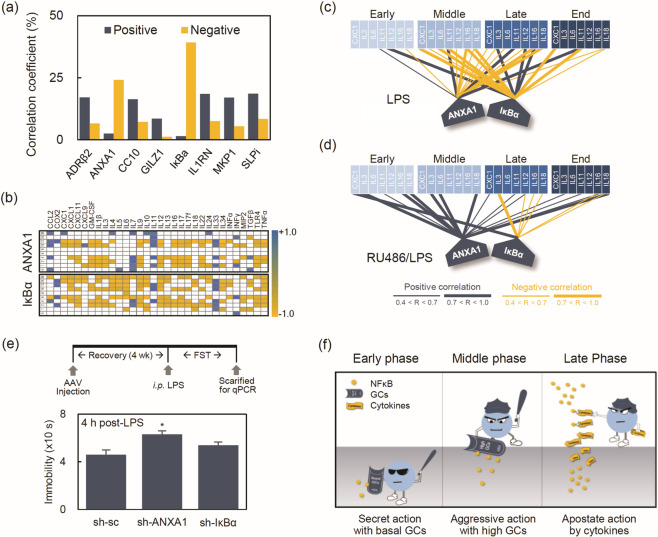
Transcriptional analysis of NFκB-GR interplay with qPCR. **a** Percentages of correlation coefficient in NFκB-GR transcriptional interplay. **b** Time-lapse heat map of the correlation coefficients of 32 NFκB-response genes with ANAX1 and IκBα (*n* = 8/group). Temporal network connectivity of (**c**) LPS-treated mice (*n* = 8/group) and (**d**) RU486/LPS-treated mice [*n* = 8/group except for 12 h post-LPS (*n* = 7/group)] with four periods. Periods were grouped according to the behavioral outcomes, and seven NFκB-responsive genes were selected for a clear correlation with ANAX1 and IκBα in the middle phase. The connecting lines are colored according to their correlation direction and specificity, as in the insert. **e** Experimental procedure and immobilization analysis of FST for AAV-based gene silencing in IL-PFC. **f** Illustrations of the proposed model. GCs, glucocorticoids, seven NFκB-responsive genes; CXC1, IL3, IL6, IL11, IL12, IL16, and IL18. In correlation analysis, only the significant correlation coefficient (*P* < 0.05) of GraphPad Prism (GraphPad Prism software v7.04, Inc.) was used. Statistical significance was determined by Student’s *t*-test (*P < 0.05 vs control for immobility analysis) (color figure online).

To gain transcriptional insight into the GR inhibitory action on NFκB in the middle phase, PCR analysis was used to investigate the negative correlation coefficients between eight GR-specific genes known to inhibit NFκB signaling [29–34] and 32 NFκB-response genes (Supplementary Table 1). Of the eight GR-specific genes, GILZ expression significantly increased in the middle phase, but the other genes did not (Supplementary Fig. 9a). This might have resulted from individual variations due to mainly endpoint analysis. Correlation analysis with the same individual mice can increase statistics significance by reducing at least intra-individual variability. As shown in Fig. 4a, the correlation coefficient showed that the two GR-specific genes encoding for ANXA1 (24.17%) and IκBα (39.27%) were mostly negatively correlated. A time-lapse heat map with ten-time points also showed a negative correlation coefficient mainly in the ANXA1 and IκBα (Fig. 4b and Supplementary Fig. 10), but unexpectedly negative correlations spread across all periods. In an alternative analysis of four periods, however, negative correlation coefficients with seven NFκB-responsive genes were found primarily in the middle phase (Fig. 4c). These negative correlations were mostly converted to positive correlations by RU486 treatment (Fig. 4d) in the middle phase. These results suggest that two GR-specific genes, ANXA1 and IκBα, are involved in protecting depressive-like behavior through inhibition of NFκB during the body-to-brain inflammatory spread.

### Effect of AAV-mediated gene silencing in the IL-PFC on depressive-like behavior by LPS

We employed RNA interference to confirm the causative association of ANAX1 or IκBα with the depressive-like behavior. After candidate shRNAs were confirmed by in vitro fluorescence and western bolt analyzes (Supplementary Fig. 11), the right IL-PFC of mice were unilaterally injected with AAV-scramble-shRNA, AAV-ANAX1-shRNA, or AAV-IκBα-shRNA vectors. Four weeks later, mice were *i.p.* injected with LPS and then the immobility in the FST was analyzed at 4 h post-LPS. Similar to the depressive-like behavior with RU486/LPS treatment, the silencing of ANAX1 increased immobility at 4 h post-LPS (Fig. 4e), indicating that ANXA1 is a key transcript in IL-PFC for protection against neuroinflammation induced by systemic LPS [35–37]. However, no change was found in the mobility with IκBα knockdown mice (data not shown). The different behavioral outcomes between ANXA1 and IκBα may be due to the IL-PFC microenvironment with less effective ANAX1 action that suppresses neuroinflammation [38], or the insufficient IκBα knockdown with the unilateral injection that affects behavioral change. Taken together, we propose that the inhibitory action of GR on NFκB changes over time in the body-to-brain inflammatory spread (Fig. 4f and Supplementary movies 1, 2).

## Discussion

This study is the first to monitor in vivo temporal NFκB and GR activity to account for how the hypothalamic-pituitary-adrenal (HPA)-axis modulates the immune system in the body and brain [39, 40]. The middle and late GRs were differentially involved in the inhibitory action on NFκB activity by a systemic inflammatory challenge; upon binding to high levels of GC, the middle-phase GR effectively inhibits NFκB, whereas the late-phase GR has no inhibitory action. Experiments with RU486 also revealed a cryptic mechanism that basal GC plays a protective role in the early brain inflammatory assault. Furthermore, by assessing gene silencing in the IL-PFC, we showed that ANAX1 plays an important role during brain inflammatory adaptation [41, 42].

A notable finding in our study was the activation of NFκB, which serves as a biological marker of the behavioral outcome. The most difficult riddle in behavioral research is linking signal activity with behavioral outcomes. Particularly, the behavioral relevance of in vivo NFκB activity is not well known. Our results of behavioral-outcome-based NFκB transition demonstrated that body and brain NFκB activation corresponded to sickness and depressive-like behaviors, respectively. This suggests a tissue-specific association with NFκB activation [43]. This depressive-like association was corroborated using RU486 experiments, in which NFκB activation and consequent depressive-like behavior appeared in the early phase.

Another finding was the temporal involvement of the GR inhibitory action on NFκB. Given the inflammation and hypercortisolism observed in depressed patients [44, 45], it is important to evaluate the impairment of the GR inhibitory function on NFκB, a neurobiological mechanism for the depressive disorder [1]. From the in vivo NFκB-GR trajectory, we suggest that the HPA-axis plays a critical role in the temporal ability of GR to inhibit NFκB activity. For example, the middle-phase GR involved in normal behavior has been shown to remarkably inhibit NFκB through GR binding to high levels of CORT [46, 47]. In contrast, the late GR involved in depressive-like behavior is considered devoid of inhibitory as evidenced by the coexistence of GR and NFκB activation in the late phase. The loss of inhibitory action can be explained by GC resistance mediated by the selective accumulation of GRβ protein induced by TNF-α treatment [22]. However, we cannot rule out the possibility of direct antagonism of NFκB by GR through protein–protein interactions. This is because in vitro TNFα treatment significantly increases GR nuclear translocation and its corresponding activity [23, 24, 48, 49]. In addition, TNFα-activated NFκB was abolished by treatment with 100 nM CORT in the H19-7 cells (Fig. 2g). This was confirmed by in vivo experiment with *i.p*. CORT injection, showing alleviated depressive-like behaviors in the late phase (Supplementary Fig. 6).

We also discovered the latent role of the basal HPA-axis in inhibitory action on NFκB activity by blocking GR activity with RU486 pretreatment in the early phase. Clinical evidence on the basal HPA-axis [50], knowing the psychiatric significance of basal GR activity can help determine why the basal HPA-axis is sometimes distorted, leading to stress-related disorders, such as depression [51–53]. Thus, our study may provide unique insights on how to direct the HPA-axis of a depressed patient to cure the antidepressant-resistant depression caused by neuroinflammation.

One challenge faced in this study was the readouts of transcription results. The nonsignificant changes in the expression levels of GR-specific genes including genes modulating GR activity (i.e., GR, SGK1, and FKBP5), were expected to be upregulated during the middle phase; however, there were no changes except for GILZ expression. This could be explained by the endpoint PCR analysis and diverse transcriptional reactivity derived from various cells in the IL-PFC. Nevertheless, other possibilities, such as the decoupling of transcript expression and GR activity could not be ruled out, as described in our previous study in which fluoxetine had no effect on hippocampal GR protein expression for GR activation [1]. Another challenge was the different statistical significance between gene expression and network analyzes. Although network analysis showed negative correlation coefficients with ANXA1 and IκBα in the middle phase, the gene expression levels were not increased. This could be explained by the statistical rigor, which could be performed using data from single individuals in the network analysis. The baseline is used as the value of interest but is considered noise in gene expression analysis. Also, similar values with gene expressions between groups are expected to perform different functions in the IL-PFC, particularly those with high individual variation. Finally, we also encountered certain difficulties in the readouts of transcriptional trajectory. The signal trajectory obtained with the reporter was clearly visible; however, it was difficult to obtain a clear trajectory with the GR specific genes in qPCR analysis. This may have been related to the different features of the detection method for signals and transcripts. Signals were monitored in a longitudinal analysis with reporters based on simple consensus sequences, generating a homogeneous trajectory. However, the endogenous promoter of the target gene is composed of various transcription factor (TF) binding elements [54, 55], resulting in complex changes in the spatiotemporal expression of the target gene [56]. Various cell types are also involved in the diversity of gene expression [57, 58]. Naturally, the results of in vivo transcripts provide more physiologically relevant information; however, as we observed in our study, such data could increase the complexity of interpreting the link between transcriptional outcomes and behavioral phenotypes. The use of our reporter, which removed this complexity, clarified signal links.

The controversial issue was the difference in the reading of phenotypic outcomes in the same behavioral rodent test between research laboratories. For example, the immobility of FST has been widely used as a parameter to measure behavioral despair and helplessness as a parameter [59]; however, it has also been suggested as habituation [60, 61] or coping with the inescapable stressor [62, 63]. This may be due to the complex features of human depression patients [64, 65] and thereby the lack of equivalence between animal models and depression [66]. In addition, the parameters of behavioral testing are known to be affected by strain, housing conditions, animal handling [67]. In antidepressant studies, the immobility of FST using ICR mice has been demonstrated as a depressive-like behavioral parameter, as evidenced by decreased immobility by fluoxetine (Supplementary Fig. 12) [64]. In contrast, OFT can be used to assess anxiety, exploration, and locomotor activity. These phenotypic outcomes can be discerned using different parameters such as the mouse’s tendency to avoid the central areas as anxiety-like behaviors [68] and total distance traveled in the OFT for a parameter of motor activity that is interpreted as a sickness behavior [69]. Nevertheless, caution should be exercised when interpreting behavioral outcomes, as the parameters of the behavioral test may be inaccurate or outdated, as suggested by the de Kloet’ group [62, 63].

The inability to investigate GR phosphorylation is a technical limitation; we could not analyze GR phosphorylation due to the discontinuation of commercial antibodies. Future studies should use antibodies for these two distinct GRs, particularly within its N-terminus (S203 and S211) [1, 70, 71]. Information on GR phosphorylation can provide detailed information on the molecular mechanisms underlying GR activation and different inhibitory GR actions on NFκB. Nevertheless, the technical strength of our study emanates from the high temporal resolution of the Luc2CP reporter, which allowed us to discern different NFκB responses between the dermis and IL-PFC during the early-phase. Analyzes using the reporters herein allow an integrated interpretation of the efficacy of drugs without further experimentation, such as the effects of RU486 on GR activity in the brain. The reporters can also be applied to a one-step drug assay, which is a simple method for testing drug effectiveness in the brain and is best suited for conceptual medical research.

In summary, this study sheds light on the role of the HPA-axis as an origin of inflammation-related depression. This information can be useful for guiding treatment by providing insight into how antidepressant-resistant depression occurs in the dysfunctional HPA-axis of depressed patients. Our in vivo signal-transition assay also provides a technical framework to elucidate how abnormalities in signaling pathways can lead to various psychiatry disorders.

## Supplementary information

Supple information

Proposed model of different NFκB responses in the brain and body

Proposed model of temporal GR inhibitory action on NFκB in the brain
