# Supplementary material for: Decoding the temporal nature of brain GR activity in the NFκB signal transition leading to depressive-like behavior
Source: Mol Psychiatry. 2021 Jan 22;26(9):5087–96. doi: 10.1038/s41380-021-01016-1 (PMC7821461; doi:10.1038/s41380-021-01016-1)
Supplement: Supplementary file 1 — Supple information [file 41380_2021_1016_MOESM1_ESM.docx]

**Full Materials and Methods**

*Construction of lentiviral-based reporters*

For the NFκB-Luc2CP reporter, the 5×GRE-adenovirus intermediate early promoter was replaced with a DNA fragment of 6×NFκB response elements-TATA box at the EcoR I and Nde I sites of the GRE-Luc2CP reporter. The DNA fragment was inserted by annealing two single-stranded oligonucleotides with complementary sequences containing the response elements, TATA box, and corresponding restriction enzyme sites (Bioneer Co., Daejeon, Korea). The EF1α-Luc2CP reporter, a positive control for signal reporters, was produced by replacing Luc with Luc2CP in the EF1α-Luc reporter. Restriction enzyme maps confirmed the lentiviral constructs in agarose gels by sequencing the ligated regions (Bioneer Co., Daejeon, Korea). The lentivirus was commercially produced at KIST Virus Facility (http://virus.kist.re.kr) and also validated in H19-7 neuronal cells (Supplementary Figure1). The titers of lentivirus were in the range of 1.50–1.95×10^12^ genome copies/ml.

*Cell culture and luciferase activity*

Hippocampal progenitor H19-7 cells, mouse microglia BV2 cells, human normal skin fibroblast Detroit 551 cells, human embryonic kidney 293T cells, and human liver cancer HepG2 cells were cultured in Dulbecco’s modified eagle medium (Hyclone, Logan, UT, USA) supplemented with 10% fetal bovine serum (Hyclone), 25 U/ml penicillin, and 25 μg/ml streptomycin at 37 ℃ in 5% CO_2_. To determine the activity of luciferase, cells were plated at a density of 10^3^ cells/well in 96-well cell culture plates and incubated for 24 h; cells were then treated with either corticosterone (Sigma), TNFα (Sigma), or RU486 (Sigma) and incubated for 36 h. Then, luciferase activity was detected using IVIS 2000.

*Animal care*

In this study, we used five-weeks-old ICR male mice (Dooyeol Biotech, Korea). The animals were individually housed in transparent plastic cages with wire grid covers and controlled temperatures (24 ± 2 ℃) with a constant 12:12 h light/dark cycle. The mice were given at least 1 week to adapt to their environment before the experiments. The animal study was approved by the Institutional Animal Care and Use Committee at the Korea Basic Science Institute (KBSI-AEC-1817). All animal procedures were carried out in accordance with the Guide for the Care and Use of Laboratory Animal Resources Commission of KBSI.

*Experimental design*

1. *Administration of lipopolysaccharide (LPS) and RU486 as an animal model*

LPS and Mifepristone were purchased from Sigma Aldrich (MO, USA). In this study, we used LPS to induce neuroinflammation and Mifepristone (known as RU486, 10 mg/kg) to antagonize corticosterone action competitively at the receptor level. Animals were divided into three groups as follows: normal control mice without any intervention except saline, LPS group which received LPS administration, and the RU486 pre-treated group, which received RU486 1 h before LPS treatment. All drugs were administrated via intraperitoneal (*i*.*p*.) injection. Sickness or depressive-like behaviors were analyzed by observations at 0, 1, 2, 4, 6, 8, 10, 12, 24, and 36 h following systemic LPS administration. Immediately following behavioral tests, mice were anesthetized with 2.5% isoflurane in 100% oxygen at a flow rate of 1.0 l/min. Blood was collected at euthanasia, followed by centrifugation of blood at 3,000 rpm for 15 min to obtain serum. Tissues were dissected, including the IL-PFC region of the brain, dermis of the back, and liver. Half of these samples were fixed in 10% buffered formalin overnight and prepared as frozen tissue slides. The remaining half was frozen in liquid nitrogen and stored at -80 ℃ until further molecular analysis.

1. *Brain stereotaxic surgery and intradermal injection for the dual-monitoring of NFκB or GR luciferase activity*

The mice were anesthetized in a chamber with 2.5% isoflurane in 100% oxygen at a flow rate of 1.0 l/min and placed in a stereotaxic apparatus (Kopf Instruments, CA, USA) with an anesthetic mask (1.0% isoflurane in 100% oxygen at 0.5 l/min). The lentiviral luciferase reporters (5xGRE-Luc2CP, 6xNFκB-Luc2CP, and EF1α-Luc2CP; titer 1.69 x 10^12^) were injected into the right IL-PFC based on coordinates from the bregma and dura mater according to the Allen Mouse Atlas (Lein et al., 2007) (anterior + 2.5 mm, lateral - 0.4 mm, ventral - 3.1 mm). A 1 μl viral solution was injected at a rate of 0.25 μl/min using a micro-syringe nanopump (Leica Microsystems, Canada) and a Hamilton syringe (33 G, 5 μl). The syringe was held in place for 10 min before withdrawal. To check the luciferase activity of the skin, we identified an appropriate site on the inner surface (dermis) of the upper back. Injection sites were free from lesions, rashes, moles, hair, or scars. The mice were injected with lentiviral luciferase reporters following the same procedure as in the IL-PFC; however, for intradermal injections, a 1 μl viral solution and Hamilton syringe (12 G, 10 μl) were used.

1. *Imaging of in vivo luciferase activity*

The bioluminescence technique for *in vivo* visualization of luciferase activity was performed according to our previous work. Fifteen minutes before every BLI acquisition, all mice received 150 mg/kg D-luciferin (Biosynth International, IL, USA) dissolved in Dulbecco’s PBS via *i*.*p*. injection, and then the mice were anesthetized in a chamber with 2.5% isoflurane in 100% oxygen at a flow rate of 1.0 l/min for 10 min. For *in vivo* BLI analyses, the mice were simultaneously exposed for 1 min to IVIS with a 2.0% mixture at a 0.5 l/min flow rate, and the regions of interest were quantified with photon flux (p/s) using Living Image software v4.2 (Xenogen Corporation). For the monitoring of *in vivo* GR or NFκB activity, mice injected with each lentiviral reporter were subjected to LPS or LPS with RU486 administration and monitored at 2 h intervals following the timeline outlined in the experimental procedure. The data represent BLI signals from the individual mice, combining at least three different studies. Although BLI is highly sensitive and has a high signal–noise ratio, the value of 3.0 × 10^4^ p/s is similar to the background signal levels. Thus, we excluded luminescent signal data that did not reach 3.0 × 10^4^ p/s. The quantified BLI for 5xGRE-Luc2CP or 6xNFκB-Luc2CP were normalized to the average BLI of an EF1α-Luc2CP control reporter due to an account for the temporal increase of luciferase activity. The timeline of the experimental procedures is presented in Supplementary Figure 2.

1. *Administration of LPS with corticosterone as an animal model*

LPS and corticosterone were purchased from Sigma Aldrich (MO, USA). In this study, we used LPS (1 mg/kg) and corticosterone (10 mg/kg). Animals were divided into two groups as follows; LPS group which received LPS administration, and the corticosterone treated group which received corticosterone 8 h after LPS injection. All drugs were administrated via intraperitoneal (*i*.*p*.) injection. Depressive-like behavior was analyzed by observations at 0, 8, 10, 12, 14, 16 h following LPS administration.

1. *Microinjection of RU486 in the IL-PFC*

The mice were anesthetized in a chamber with 2.5% isoflurane in 100% oxygen at a flow rate of 1.0 l/min and placed in a stereotaxic apparatus (Kopf Instruments, CA, USA) with an anesthetic mask (1.0% isoflurane in 100% oxygen at 0.5 l/min). The implantation of the Neuroscience guide-cannula 26-gauge (WPI, FL, USA) into the IL-PFC was performed. The coordinates for implantation of the guide-cannula were an angle 0° (anterior + 2.5 mm, lateral – 0.4 mm, ventral -3.1 mm) for targeting IL-PFC. To help anchor the guide-cannula, two screws were anchored in the skull to the rear side of the implantation site of the guide-cannula. Carboxylate dental cement (Durelon, 3M, St.Paul, MN) was applied to the surface of the skull around the base of the cannula to fix the guide-cannula on the skull. After implantation, mice were given a subcutaneous injection of saline for four weeks [1, 2]. Four weeks after surgery, animals received microinjection of RU486 (1 ng) and *i.p.* LPS (1mg/kg) administration. Depressive-like behavior was analyzed by observations at 1, 4, and 8 h post-LPS.

*AAV-based gene silencing in the IL-PFC*

1. *Plasmids and shRNAs*

cDNAs encoding full-length mouse ANXA1 and IκBα (GenBank accession no. NM_010730 and NM_010907) were synthesized from Integrated DNA Technologies (IDT, Coralville, IA) and cloned into pDONR207 vector using Gateway BP clonase II Enzyme mix (Thermo Fisher, Waltham, MA). These two entry clones were converted to an expression vector containing pDEST-GFP-N or pDEST-GFP-C by a gateway LR clonase II mix (Thermo Fisher, Waltham, MA), which was subsequently used to verify shRNA efficiency. Using the BLOCK-ITTM RNAi Designer program, we selected 3 candidate sequences (ANXA1 shRNA1: 5’- gctgccttgcacaaagctatc - 3’; ANXA1 shRNA2: 5’- gccatgaagggacttggaaca - 3’; ANXA1 shRNA3: 5’ - ggacttgagtgtgaatcaaga - 3’; IκBα shRNA1: 5’- ggtgactttgggtgctgatgt - 3’; IκBα shRNA2: 5’- gtggagcacttggtgactttg - 3’; IκBα shRNA3: 5’ - ggaattgctgaggcacttctg - 3’) each to silence the expression of ANXA1 or IκBα. These sequences were inserted in the pAAV-SicoR vector [3], with two loxP sequences removed for stable expression *in vivo*. Knockdown efficiency of the three candidate shRNAs was evaluated by Western blotting with the lysate of cells co-transfected with GFP-tagged target gene expression vector and each shRNA AAV vectors in HEK293T cells.

1. *Production of shRNA Adeno-Associated Virus*

After testing the three candidate shRNAs, one shRNA with the highest knockdown efficiency was constructed with recombinant AAV for *in vivo* experiments for ANXA1 and IκBα. AAVs (serotype DJ) were produced using a previously described three-plasmid cotransfection system [4]. The production and purification of AAVs were carried out at the KIST Virus Facility (http://virus.kist.re.kr). A scrambled shRNA AAV with a non-target sequence was produced together as a control.

1. *Brain stereotaxic surgery for the AAV based shRNAs*

The mice were anesthetized in a chamber with 2.5% isoflurane in 100% oxygen at a flow rate of 1.0 l/min and placed in a stereotaxic apparatus (Kopf Instruments, CA, USA) with an anesthetic mask (1.0% isoflurane in 100% oxygen at 0.5 l/min). The AAVs (scramble shRNA-mCherry, ANXA1 shRNA-mCherry, and IkBa shRNA-mCherry; titer 1.0 x 10^12^) were injected into the right IL-PFC based on coordinates from the bregma and dura mater according to the Allen Mouse Atlas (Lein et al., 2007, anterior + 2.5 mm, lateral – 0.4 mm, ventral -3.1 mm). A 1 ul viral solution was injected at a rate of 0.25 ul/min using a micro-syringe nanopump (Leica Microsystems, Canada) and a Hamilton syringe (33 G, 5 ul). The syringe was held in place for 10 min before withdrawal. Four weeks after stereotaxic injection, depression-like behavior was analyzed at 4 post-LPS. Immediately following behavioral tests, brain tissue was collected for RT-PCR and immunohistochemical staining.

*Behavioral analysis*

For behavioral testing, sample size was chosen based on previous experience and standards in the field.

1. *Open field test (OFT)*

The OFT was carried out in a square arena (40 cm x 40 cm x 40 cm) with a clear Plexiglas floor and walls placed inside an isolation chamber with dim illumination. Mice were placed in the center of the box and allowed to freely explore the arena undisturbed for 10 min. Mice were videotaped using a camera fixed above the arena. Video analysis and data acquisition were obtained with a video-tracking system (EthoVision) to analyze total distance, mean velocity, and time spent in the center of the arena. A subset of the data sets had been analyzed using a double-blind approach, resulting in the same distributions as in the non-blinded analysis.

1. *Forced swim test (FST)*

Mice were placed in a clear glass cylinder (height: 20 cm, diameter: 12 cm) containing approximately 15 cm of water (22 ± 1 ℃) so they could neither escape nor reach the bottom. The apparatus was portioned so that animals were unable to observe animals in the neighboring cylinders. Mice were forced to swim for 7 min. The animals were habituated for the first 1 min, and behavior was monitored over the next 6 min and scored by time of immobility. The investigators performing the time of immobility analysis were unaware of the sample identity.

*Measurement of plasma CORT and IL1β*

Blood was collected during the following experiments via a cardiac puncture into heparinized collection tubes, then spun down, and plasma was stored at –80 ℃ until further analysis. The levels of CORT and IL1β were quantified by Corticosterone (ENZO, NY, USA) and Mouse IL1β (R&D Systems, MN, USA) ELISA kits. The ELISA assays were performed following the instructions of the manufacturer.

*Immunofluorescence and confocal imaging*

The tissues were collected and post-fixed in 4% paraformaldehyde overnight at 4 ℃. The samples were embedded in ovine trophoblastin protein (OTP) solutions for cryo-block, coronally sectioned to a thickness of 30 μm and processed for immunofluorescence staining with primary and secondary antibodies specific for GR (1:200; SC-1004, Santa Cruz Biotechnology, TX, USA), NeuN (1:100; MAB377, EDM Millipore MA, USA), IBA-1 (1:500; LKL0566, Wako, VA, USA), GFAP (1:1000; ab7260, United Kingdom) and Keratinocyte (1:100; ab52635, Abcam, United Kingdom). Cells were fixed for 30 min at room temperature with 4 % formaldehyde. The fixed cells were then washed with PBS and incubated at room temperature for 2 h in PBS containing 10 % BSA and 0.5 % Tween-20. Cell staining was carried out with primary antibodies specific for GR (1:200; SC-1004, Santa Cruz Biotechnology, TX, USA), NFκB p65 (1:400; 6956, Cell Signaling, MA, USA) and SC-35 (1:200, SC-53518, Santa Cruz Biotechnology, TX, USA), Donkey anti-Rabbit Alexa 488 (1:400, A-21206, Thermo Fisher Scientific, MA, USA), Donkey anti-Rabbit Alexa 594 (1:400, A-21207, Thermo Fisher Scientific, MA, USA), Donkey anti-Mouse Alexa 594 (1:400, A-21203, Thermo Fisher Scientific, MA, USA), Donkey anti- Mouse Alexa 488 (1:400, A-21202 Thermo Fisher Scientific, MA, USA). Analysis of the nuclear translocation of GR, p65, and SC-35 proteins was performed by measuring at least 10 different points within each image for each protein. The images were taken with an inverted fluorescence confocal laser-scanning microscope (TCS SP8 STED, Leica Microsystems, Germany) equipped with a 63x/1.40 W objective lens.

*Real-time polymerase chain reaction (PCR) analysis*

Total RNA was extracted from the IL-PFC of the brain, dermis of the back, and liver using Trizol reagent (Invitrogen, USA) following the instructions of the manufacturer. The cDNA was prepared using a Quanti Nova Reverse Transcription kit (Qiagen, MD, USA). Quantitative PCR was performed on Step-One Plus (Applied Biosystems, MA, USA) using an SYBR green PCR kit (Bioneer, Korea) and gene-specific primers, 0.05 μg cDNA samples were used with 40 cycles of amplification. Relative quantitation for PCR products was normalized to β-actin as an internal standard. The PCR primer sequences are listed in Supplementary Table 1.

Table 1. Primers used in real-time PCR

| Gene Abbrev. | Gene Name | Sequences of Primers (5'-3') | Reference |
| --- | --- | --- | --- |
| *ADRβ2* | Adrenergic receptor, beta 2 | F: GGGAACGACAGCGACTTCTT  R: GCCAGGACGATAACCGACAT | [5] |
| *ANXA1* | Annexin A1 | F: ATGTATCCTCGGATGTTGCTGC  R: TGAGCATTGGTCCTCTTGGTA | [6] |
| *Actb* | Actin, beta | F: CCAACCGTGAAAAGATGACC  R: CCAGAGGCATACAGGGACAG | [7] |
| *CC10*  *(Scgb1a1)* | secretoglobin, family 1A, member 1 | F: ATGAAGATCGCCATCACAATCAC  R: GGATGCCACATAACCAGACTCT | [8] |
| *CCL2* | chemokine (C-C motif) ligand 2 | F: GGCCTGCTGTTCACAGTTGC  R: CCTGCTGCTGGTGATCCTCTT | [9] |
| *COX2*  *(Ptgs2)* | prostaglandin-endoperoxide synthase 2 | F: TGAAGACGTCCTCCACTCATG  R: CCTGGGATGGCATCAGTTT | [10] |
| *CXCR1* | chemokine (C-X-C motif) receptor 1 | F: ACTGCACCCAAACCGAAGTC  R: CAAGGGAGCTTCAGGGTCAA | [11] |
| *CXCL1* | chemokine (C-X-C motif) ligand 1 | F: GACCATGGCTGGGATTCACC  R: CCAAGGGAGCTTCAGGGTCA | [12] |
| *CXCL9* | chemokine (C-X-C motif) ligand 9 | F: CCGAGGCACGATCCACTACA  R: CGAGTCCGGATCTAGGCAGGT | [12] |
| *CXCL11* | chemokine (C-X-C motif) ligand 11 | F: CTGCACAGATGAGAGACAAATTCC  R: GAAGCTGCAAAGATCCCAATG | Designed by Primer bank |
| *CXCL13* | chemokine (C-X-C motif) ligand 13 | F: TTCTGGAAGCCCATTACACAAA  R: CCATTTGGCACGAGGATTCA | [13] |
| *FKBP4* | FK506 binding protein 4 | F: CCTCTCGAAGGAGTGGACATC  R: TCCCCGATCATGGGTGTCT | [14] |
| *FKBP5* | FK506 binding protein 5 | F: TGAGGGCACCAGTAACAATGG  R: CAACATCCCTTTGTAGTGGACAT | [15] |
| *GAPDH* | glyceraldehyde-3-phosphate dehydrogenase | F: CAAGGTCATCCATGACAACTTTG  R: GGCCATCCACAGTCTTCTGG | [16] |
| *GILZ1 (Tsc22d3)* | TSC22 domain family, member 3 | F: AACACCGAAATGTATCAGACCC  R: GTTTAACGGAAACCAAATCCCCT | [17] |
| *GM-CSF (Csf2)* | colony stimulating factor 2 (granulocyte-macrophage) | F: CGTTCC CCTGGTCAGTGTC  R: CCGCTGGCCTGGATCTTC | Designed by Primer bank |
| *GR*  *(Nr3c1)* | nuclear receptor subfamily 3, group C, member 1 | F: ATCATACAGACAAGCAAGTGGAA  R: AGGGTAGAGTCATTCTCTGCTC | [18] |
| *IFNa* | interferon alpha | F: AGCCACGGAGAGTCAATGG  R: GCTCTGACACGAAACTGTGTTTT | [19] |
| *IFNg* | interferon gamma | F: GTAAGAGGAGCAACCACCAGAA  R: GACGTCTGTATCCCTCCTTTCC | [20] |
| *IκBα (nfkbia)* | nuclear factor of kappa light polypeptide gene enhancer in B cells inhibitor, alpha | F: TGAAGGACGAGGAGTACGAGC  R: TTCGTGGATGATTGCCAAGTG | [21] |
| *IL1β* | interleukin 1 beta | F: GGAAGGTCCACGGGAAAGAC  R: AGGCAGGCAGTATCACTCATTGT | [22] |
| *IL1RN* | interleukin 1 receptor antagonist | F: GCTCATTGCTGGGTACTTACAA  R: CCAGACTTGGCACAAGACAGG | [23] |
| *lL3* | Interleukin 3 | F: CTGCCTACATCTGCGAATGACT  R: CAGATCGTTAAGGTGGACCATG | Designed by Primer bank |
| *IL4* | Interleukin 4 | F: GAAGCCCTACAGACGAGCTCA  R: ACAGGAGAAGGGACGCCA | [24] |
| *lL5* | Interleukin 5 | F: ACCGAGCTCTGTTGACAAG  R: TCCTCGCCACACTTCTCTTT | Designed by Primer bank |
| *IL6* | Interleukin 6 | F: GAGGATACCACTCCCAACAGACC  R: AAGTGCATCATCGTTGTTCATACA | [25] |
| *IL7* | Interleukin 7 | F: TTCCTCCACTGATCCTTGTTCT  R: AGCAGCTTCCTTTGTATCATCAC | [26] |
| *IL9* | Interleukin 9 | F: ATGTTGGTGACATACATCCTTGC  R: TGACGGTGGATCATCCTTCAG | [27] |
| *IL10* | Interleukin 10 | F: ACCTGCTCCACTGCCTTGCT  R: GGTTGCCAAGCCTTATCGGA | [28] |
| *IL11* | Interleukin 11 | F: TGTTCTCCTAACCCGATCCCT  R: CAGGAAGCTGCAAAGATCCCA | [29] |
| *lL12* | Interleukin 12 | F: CAGAAGCTAACCATCTCCTGGTTTG  R: -TCCGGAGTAATTTGGTG CTTCACAC | [30] |
| *IL16* | Interleukin 16 | F: AAGAGCCGGAAATCCACGAAA  R: GTCTCAAAAGGGTCAGGGTACT | [31] |
| *IL17* | Interleukin 17 | F: GTGTCAATGCGGAGGGAA  R: TTCAGGACCAGGATCTCTTGCT | [32] |
| *IL17F* | Interleukin 17F | F: TGCTACTGTTGATGTTGGGAC  R: AATGCCCTGGTTTTGGTTGAA | [33] |
| *IL18* | Interleukin 18 | F: CACATGCGCCTTGTGATGAC  R: TGCAGCCTGGGGTATTCTGT | [34] |
| *IL22* | Interleukin 22 | F: ATGAGTTTTTCCCTTATGGGGAC  R: GCTGGAAGTTGGACACCTCAA | [35] |
| *IL24* | Interleukin 24 | F: GAGCCTGCCCAACTTTTTGTG  R: TGTGTTGAAGAAAGGGCCAGT | [36] |
| *IL33* | Interleukin 33 | F: TCCAACTCCAAGATTTCCCCG  R: CATGCAGTAGACATGGCAGAA | [37] |
| *IL34* | Interleukin 34 | F: TTGCTGTAAACAAAGCCCCAT  R: CCGAGACAAAGGGTACACATTT | [38] |
| *CXCL2* | chemokine (C-X-C motif) ligand 2 | F: AGCCCCCCTGGTTCAGAA  R: GCTCCTCCTTTCCAGGTCAGT | [39] |
| *MKP1 (Dusp1)* | dual specificity phosphatase 1 | F: GTTGTTGGATTGTCGCTCCTT  R: TTGGGCACGATATGCTCCAG | [40] |
| *SGK1* | serum- and glucocorticoid-inducible kinase | F: GAGAAGGATGGGCCTGAACGAT  R: CGGACCCAGGTTGATTTGTTGA | Designed by Primer bank |
| *SLPi* | secretory leukocyte peptidase inhibitor | F: GGCCTTTTACCTTTCACGGTG  R: TACGGCATTGTGGCTTCTCAA | [41] |
| *TGFβ*  *(Tgfbr1)* | secretory leukocyte peptidase inhibitor | F: GTGCCCGAACCCCCATTGCT  R: CGTTTGGGGCTGATCCCGTTGAT | [42] |
| *TLR4* | toll-like receptor 4 | F: GCAGCAGGTGGAATTGTATCG  R: TGTGCCTCCCCAGAGGATT | [43] |
| *TNFa (Tnf)* | tumor necrosis factor | F: CTTCACAGAGCAATGACTCCAAAG  R: CTGAGGTCAATCTGCCCAAGTAC | [44] |

*Statistical analysis*

Results are expressed as the mean ± standard error of measure. Statistical analyses of the data were performed using Graphpad. The number of animals varied per experiment and are noted in the corresponding figure legends. The data were analyzed by one-way or two-way analysis of variance (ANOVA) tests, and the statistical significance between groups was determined by Sidak multiple comparison test. Other statistical analyses, including Student’s t-tests, are discussed under the appropriate categories in the figure legends. Data are expressed as the mean ± standard error of the mean (SEM); P < 0.05 was considered statistically significant.

**References**

1. NamKoong C, Kim MS, Jang BT, Lee YH, Cho YM, Choi HJ. Central administration of GLP-1 and GIP decreases feeding in mice. Biochem Biophys Res Commun 2017; **490**(2)**:** 247-252.

2. Groblewski PA, Cunningham CL. Repeated microinjections into the medial prefrontal cortex (mPFC) impair extinction of conditioned place preference in mice. Behav Brain Res 2012; **230**(1)**:** 299-303.

3. Shim HG, Jang SS, Kim SH, Hwang EM, Min JO, Kim HY *et al.* TNF-alpha increases the intrinsic excitability of cerebellar Purkinje cells through elevating glutamate release in Bergmann Glia. Sci Rep 2018; **8**(1)**:** 11589.

4. Huang X, Hartley AV, Yin Y, Herskowitz JH, Lah JJ, Ressler KJ. AAV2 production with optimized N/P ratio and PEI-mediated transfection results in low toxicity and high titer for in vitro and in vivo applications. J Virol Methods 2013; **193**(2)**:** 270-277.

5. Renz BW, Takahashi R, Tanaka T, Macchini M, Hayakawa Y, Dantes Z *et al.* beta2 Adrenergic-Neurotrophin Feedforward Loop Promotes Pancreatic Cancer. Cancer Cell 2018; **34**(5)**:** 863-867.

6. Zigmond E, Samia-Grinberg S, Pasmanik-Chor M, Brazowski E, Shibolet O, Halpern Z *et al.* Infiltrating monocyte-derived macrophages and resident kupffer cells display different ontogeny and functions in acute liver injury. J Immunol 2014; **193**(1)**:** 344-353.

7. Furukawa C, Fujii N, Manabe A, Matsunaga T, Endo S, Hasegawa H *et al.* Up-Regulation of Transient Receptor Potential Melastatin 6 Channel Expression by Tumor Necrosis Factor-alpha in the Presence of Epidermal Growth Factor Receptor Tyrosine Kinase Inhibitor. J Cell Physiol 2017; **232**(10)**:** 2841-2850.

8. Vaughan AE, Brumwell AN, Xi Y, Gotts JE, Brownfield DG, Treutlein B *et al.* Lineage-negative progenitors mobilize to regenerate lung epithelium after major injury. Nature 2015; **517**(7536)**:** 621-625.

9. Kalbasi A, Komar C, Tooker GM, Liu M, Lee JW, Gladney WL *et al.* Tumor-Derived CCL2 Mediates Resistance to Radiotherapy in Pancreatic Ductal Adenocarcinoma. Clin Cancer Res 2017; **23**(1)**:** 137-148.

10. Sakkou M, Wiedmer P, Anlag K, Hamm A, Seuntjens E, Ettwiller L *et al.* A role for brain-specific homeobox factor Bsx in the control of hyperphagia and locomotory behavior. Cell Metab 2007; **5**(6)**:** 450-463.

11. Zhang Y, Bi Y, Yang H, Chen X, Liu H, Lu Y *et al.* mTOR limits the recruitment of CD11b+Gr1+Ly6Chigh myeloid-derived suppressor cells in protecting against murine immunological hepatic injury. J Leukoc Biol 2014; **95**(6)**:** 961-970.

12. Liu Q, Jia J, Yang T, Fan Q, Wang L, Ma G. Pathogen-Mimicking Polymeric Nanoparticles based on Dopamine Polymerization as Vaccines Adjuvants Induce Robust Humoral and Cellular Immune Responses. Small 2016; **12**(13)**:** 1744-1757.

13. Foo SY, Zhang V, Lalwani A, Lynch JP, Zhuang A, Lam CE *et al.* Regulatory T cells prevent inducible BALT formation by dampening neutrophilic inflammation. J Immunol 2015; **194**(9)**:** 4567-4576.

14. Caldwell KE, Labrecque MT, Solomon BR, Ali A, Allan AM. Prenatal arsenic exposure alters the programming of the glucocorticoid signaling system during embryonic development. Neurotoxicol Teratol 2015; **47:** 66-79.

15. Lee SH, Johnson D, Luong R, Sun Z. Crosstalking between androgen and PI3K/AKT signaling pathways in prostate cancer cells. J Biol Chem 2015; **290**(5)**:** 2759-2768.

16. Pieper AA, Wu X, Han TW, Estill SJ, Dang Q, Wu LC *et al.* The neuronal PAS domain protein 3 transcription factor controls FGF-mediated adult hippocampal neurogenesis in mice. Proc Natl Acad Sci U S A 2005; **102**(39)**:** 14052-14057.

17. Tsai CY, Tsai CY, Arnold SJ, Huang GJ. Ablation of hippocampal neurogenesis in mice impairs the response to stress during the dark cycle. Nat Commun 2015; **6:** 8373.

18. Ishii T, Masuzaki H, Tanaka T, Arai N, Yasue S, Kobayashi N *et al.* Augmentation of 11beta-hydroxysteroid dehydrogenase type 1 in LPS-activated J774.1 macrophages--role of 11beta-HSD1 in pro-inflammatory properties in macrophages. FEBS Lett 2007; **581**(3)**:** 349-354.

19. Thompson LJ, Lai JF, Valladao AC, Thelen TD, Urry ZL, Ziegler SF. Conditioning of naive CD4(+) T cells for enhanced peripheral Foxp3 induction by nonspecific bystander inflammation. Nat Immunol 2016; **17**(3)**:** 297-303.

20. Taylor S, Mehina E, White E, Reeson P, Yongblah K, Doyle KP *et al.* Suppressing Interferon-gamma Stimulates Microglial Responses and Repair of Microbleeds in the Diabetic Brain. J Neurosci 2018; **38**(40)**:** 8707-8722.

21. Maseda D, Meister S, Neubert K, Herrmann M, Voll RE. Proteasome inhibition drastically but reversibly impairs murine lymphocyte development. Cell Death Differ 2008; **15**(3)**:** 600-612.

22. Nishimatsu H, Suzuki E, Saito Y, Niimi A, Nomiya A, Fukuhara H *et al.* Senescent Cells Impair Erectile Function through Induction of Endothelial Dysfunction and Nerve Injury in Mice. PLoS One 2015; **10**(4)**:** e0124129.

23. Fouraschen SM, Wolf JH, van der Laan LJ, de Ruiter PE, Hancock WW, van Kooten JP *et al.* Mesenchymal Stromal Cell-Derived Factors Promote Tissue Repair in a Small-for-Size Ischemic Liver Model but Do Not Protect against Early Effects of Ischemia and Reperfusion Injury. J Immunol Res 2015; **2015:** 202975.

24. Yang C, Kim SH, Bianco NR, Robbins PD. Tumor-derived exosomes confer antigen-specific immunosuppression in a murine delayed-type hypersensitivity model. PLoS One 2011; **6**(8)**:** e22517.

25. Murakami M, Okuyama Y, Ogura H, Asano S, Arima Y, Tsuruoka M *et al.* Local microbleeding facilitates IL-6- and IL-17-dependent arthritis in the absence of tissue antigen recognition by activated T cells. J Exp Med 2011; **208**(1)**:** 103-114.

26. Hou L, Jie Z, Liang Y, Desai M, Soong L, Sun J. Type 1 interferon-induced IL-7 maintains CD8+ T-cell responses and homeostasis by suppressing PD-1 expression in viral hepatitis. Cell Mol Immunol 2015; **12**(2)**:** 213-221.

27. Xue G, Jin G, Fang J, Lu Y. IL-4 together with IL-1beta induces antitumor Th9 cell differentiation in the absence of TGF-beta signaling. Nat Commun 2019; **10**(1)**:** 1376.

28. Wallgren P, Belak K, Ehlorsson CJ, Bergstrom G, Lindberg M, Fossum C *et al.* Postweaning multisystemic wasting syndrome (PMWS) in Sweden from an exotic to an endemic disease. Vet Q 2007; **29**(4)**:** 122-137.

29. Meana C, Garcia-Rostan G, Pena L, Lorden G, Cubero A, Orduna A *et al.* The phosphatidic acid phosphatase lipin-1 facilitates inflammation-driven colon carcinogenesis. JCI Insight 2018; **3**(18).

30. Kim TS, Kang BY, Cho D, Kim SH. Induction of interleukin-12 production in mouse macrophages by berberine, a benzodioxoloquinolizine alkaloid, deviates CD4+ T cells from a Th2 to a Th1 response. Immunology 2003; **109**(3)**:** 407-414.

31. Shi Y, Bollam SR, White SM, Laughlin SZ, Graham GT, Wadhwa M *et al.* Rac1-Mediated DNA Damage and Inflammation Promote Nf2 Tumorigenesis but Also Limit Cell-Cycle Progression. Dev Cell 2016; **39**(4)**:** 452-465.

32. Park E, Kim D, Lee SM, Jun HS. Inhibition of lysophosphatidic acid receptor ameliorates Sjogren's syndrome in NOD mice. Oncotarget 2017; **8**(16)**:** 27240-27251.

33. von Vietinghoff S, Ley K. IL-17A controls IL-17F production and maintains blood neutrophil counts in mice. J Immunol 2009; **183**(2)**:** 865-873.

34. Feng F, Li Z, Potts-Kant EN, Wu Y, Foster WM, Williams KL *et al.* Hyaluronan activation of the Nlrp3 inflammasome contributes to the development of airway hyperresponsiveness. Environ Health Perspect 2012; **120**(12)**:** 1692-1698.

35. Chun E, Lavoie S, Fonseca-Pereira D, Bae S, Michaud M, Hoveyda HR *et al.* Metabolite-Sensing Receptor Ffar2 Regulates Colonic Group 3 Innate Lymphoid Cells and Gut Immunity. Immunity 2019; **51**(5)**:** 871-884 e876.

36. Li X, Zhang C, Bian Q, Gao N, Zhang X, Meng Q *et al.* Integrative functional transcriptomic analyses implicate specific molecular pathways in pulmonary toxicity from exposure to aluminum oxide nanoparticles. Nanotoxicology 2016; **10**(7)**:** 957-969.

37. Nabekura T, Girard JP, Lanier LL. IL-33 receptor ST2 amplifies the expansion of NK cells and enhances host defense during mouse cytomegalovirus infection. J Immunol 2015; **194**(12)**:** 5948-5952.

38. Baek JH, Zeng R, Weinmann-Menke J, Valerius MT, Wada Y, Ajay AK *et al.* IL-34 mediates acute kidney injury and worsens subsequent chronic kidney disease. J Clin Invest 2015; **125**(8)**:** 3198-3214.

39. Zhang Y, Brenner M, Yang WL, Wang P. Recombinant human MFG-E8 ameliorates colon damage in DSS- and TNBS-induced colitis in mice. Lab Invest 2015; **95**(5)**:** 480-490.

40. Au SH, Fobel R, Desai SP, Voldman J, Wheeler AR. Cellular bias on the microscale: probing the effects of digital microfluidic actuation on mammalian cell health, fitness and phenotype. Integr Biol (Camb) 2013; **5**(8)**:** 1014-1025.

41. Iwamoto Y, Nishikawa K, Imai R, Furuya M, Uenaka M, Ohta Y *et al.* Intercellular Communication between Keratinocytes and Fibroblasts Induces Local Osteoclast Differentiation: a Mechanism Underlying Cholesteatoma-Induced Bone Destruction. Mol Cell Biol 2016; **36**(11)**:** 1610-1620.

42. Palazuelos J, Klingener M, Aguirre A. TGFbeta signaling regulates the timing of CNS myelination by modulating oligodendrocyte progenitor cell cycle exit through SMAD3/4/FoxO1/Sp1. J Neurosci 2014; **34**(23)**:** 7917-7930.

43. Smith DD, Tan X, Raveendran VV, Tawfik O, Stechschulte DJ, Dileepan KN. Mast cell deficiency attenuates progression of atherosclerosis and hepatic steatosis in apolipoprotein E-null mice. Am J Physiol Heart Circ Physiol 2012; **302**(12)**:** H2612-2621.

44. Jia L, Vianna CR, Fukuda M, Berglund ED, Liu C, Tao C *et al.* Hepatocyte Toll-like receptor 4 regulates obesity-induced inflammation and insulin resistance. Nat Commun 2014; **5:** 3878.

45. Wendeln AC, Degenhardt K, Kaurani L, Gertig M, Ulas T, Jain G *et al.* Innate immune memory in the brain shapes neurological disease hallmarks. Nature 2018; **556**(7701)**:** 332-338.

**Supplementary Figures**

**
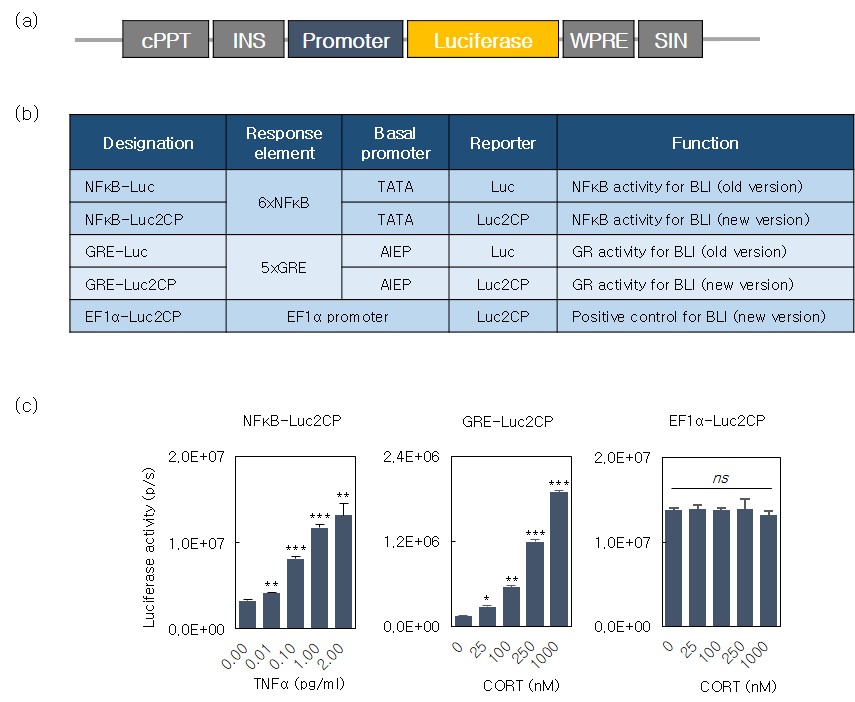
**

**Supplementary Figure 1. *In vitro* validation of the lentivirus-based reporters in the bioluminescence assay.** (a) Lentiviral reporter construct for quantitative analysis. (b) List of the reporters and their functions. (c) Responses of the reporters to TNFα or CORT in H19-7 neuronal cells. To evaluate the effects of ligand on their transcriptional activities, H19-7 cells were infected with the reporters and incubated for 36 h in the presence of indicated doses of ligands. Luciferase activity was measured using IVIS 200. (n = 3/group). Statistical significance was determined by Student’s t-test (**P* < 0.05, ***P* < 0.01, ****P* < 0.001, ns = non-significant *vs* control). cPPT, central polypurine tract; INS, chicken β-globin insulator; WPRE, woodchuck hepatitis virus posttranscriptional regulatory element; SIN, self-inactivating; TA, TATA box sequence; AIEP, minimal promoter; BLI, bioluminescent imaging.


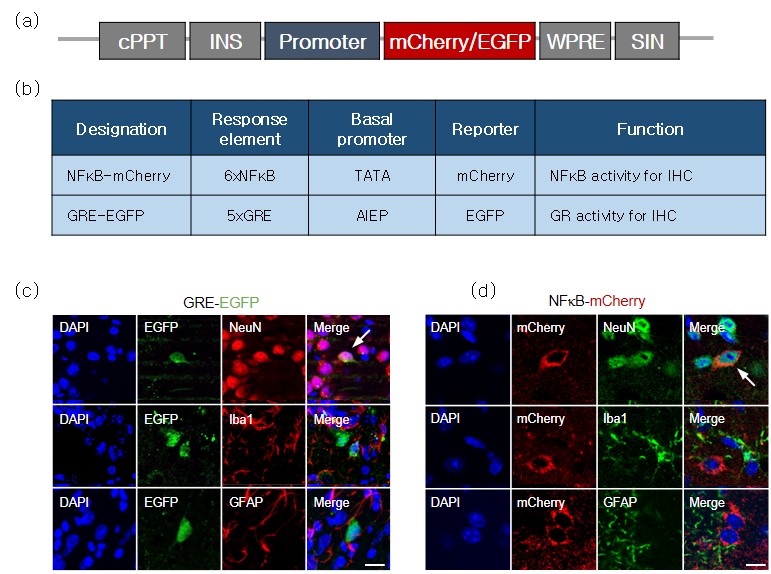
ㅇ

**Supplementary Figure 2.** **Cellular location of the signal activations in the IL-PFC using IHC assay.** (a) Lentiviral reporter construct for qualitative analysis. (b) List of the reporters and their functions. (c) Representative confocal images for GR activity. (d) Representative confocal images for NFκB activity. Four-weeks after injection, mice were subjected to LPS and sacrificed for IHC assessment at 12 h post-LPS. IL-PFC sections were stained with NeuN for neuron, Iba1 for microglia, GFAP for astrocyte and DAPI to detect the location of EGFP (green) or mCherry expression (red). As shown with arrows, GR and NFκB activations were mainly detected in NeuN^+^ neurons in which expressions of EGFP, mCherry were co-localized with GRs, NFκB respectively. Scale bar, 10 μm. cPPT, central polypurine tract; INS, chicken β-globin insulator; WPRE, woodchuck hepatitis virus posttranscriptional regulatory element; SIN, self-inactivating; TA, TATA box sequence; AIEP, minimal promoter; IHC, immunocytochemistry.


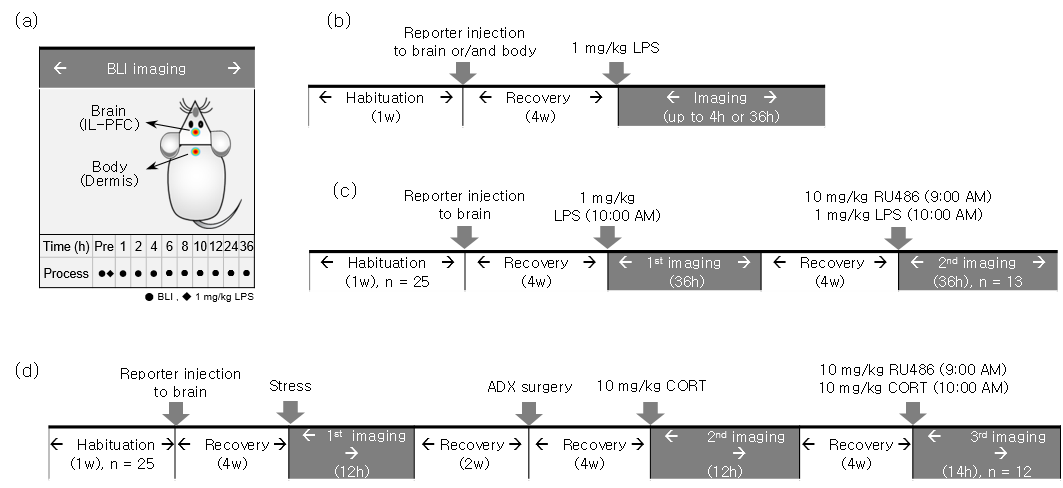


**Supplementary Figure 3. Schedule of experimental procedures.** (a) Timeline scheme for the bioluminescence where the indicated time is the time after LPS treatment (post-LPS). (b) Single- or dual-monitoring for temporal NFκB or GR activity. (c) Two repeated monitoring for temporal NFκB or GR activity. (d) Three repeated monitoring in adrenalectomized mice. The repeated-monitoring was performed to exclude possible false-negative results using the same cohort mice for the imaging process. Nevertheless, we cannot exclude the potential effects of 1st LPS treatment on the behavioral and/or neurobiological influences when mice were exposed to 2nd LPS treatment [45]. BLI, bioluminescent imaging; IL-PFC, infralimbic prefrontal cortex; LPS, lipopolysaccharide; CORT, corticosterone.


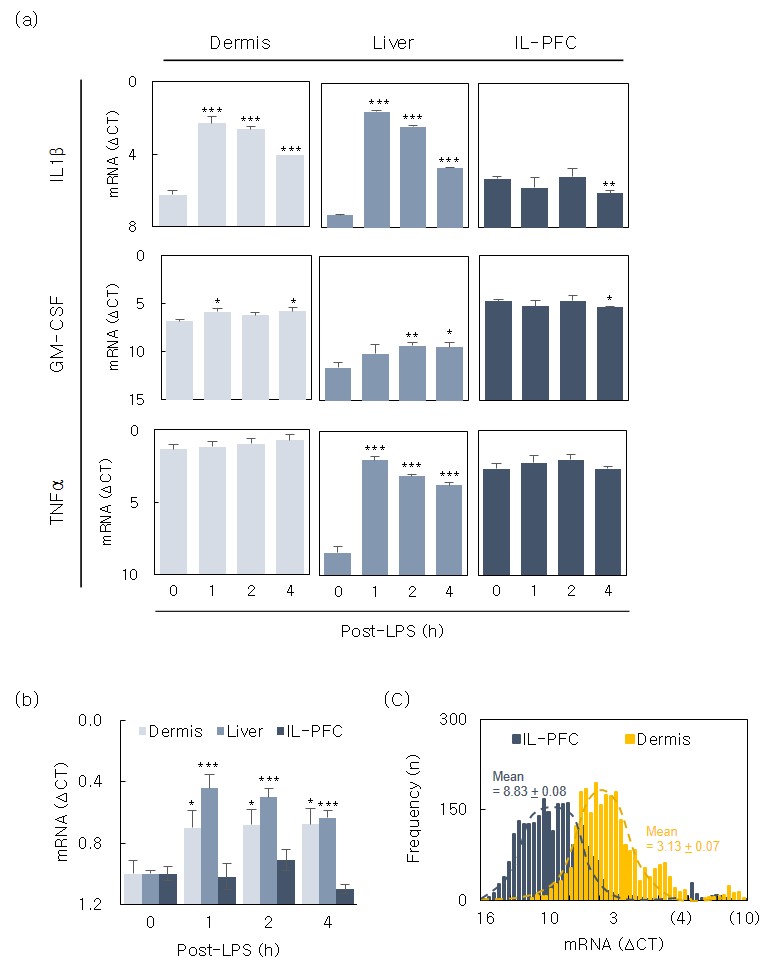


**Supplementary Figure 4.** **Time course of pro-inflammatory cytokine expression by qPCR.** (a) The expression levels of three inflammatory cytokine mRNAs (IL1β, GM-CSF and TNFα) and (b) The average combined mRNAs levels from the liver, dermis, and IL-PFC tissues. The gene expression levels were normalized to β-actin and then divided by the average of each corresponding control at 0 h post-LPS. (c) Low distribution of NFκB-driven mRNA (n = 2,560) in the IL-PFC (blue) compared to dermis (orange). Single gene expression data represent the mean of three mRNAs ± standard error of measurement (n = 5/each time point). Statistical significance was determined by Student’s t-test (**P* < 0.05, ***P* < 0.01, ****P* < 0.001 *vs* control). For the combined mRNAs analysis, two-way analysis of variance (ANOVA) indicated that there were significant interactions of IL-PFC with dermis (F_3,112_ = 9.11, *P* < 0.001) and liver (F_3,112_ = 7.47, *P* < 0.001), but there is no interaction between dermis and liver (F_3,112_ = 0.09, *P* = 0.97). Gene expression was normalized to β-actin (bin = 1.0). In the frequency analysis, a total of 32 genes were analyzed for NFκB-driven mRNA in the IL-PFC and dermis (n = 8/group). IL-PFC, infralimbic prefrontal cortex.


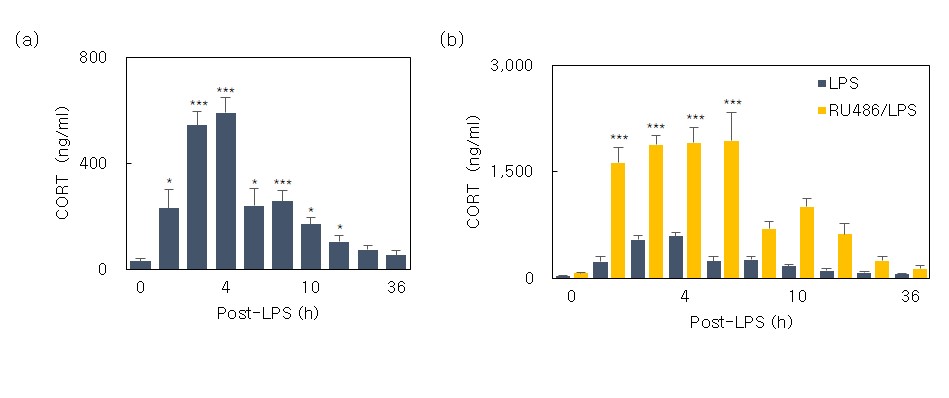


**Supplementary Figure 5.** **Time course of plasma CORT.** (a) Only LPS treatment (b) Comparison between LPS and RU486/LPS treatments (n = 8/group). Statistical significance was determined by Student’s t-test (**P* < 0.05, ****P <* 0.001 *vs* control at 0 h post-LPS)


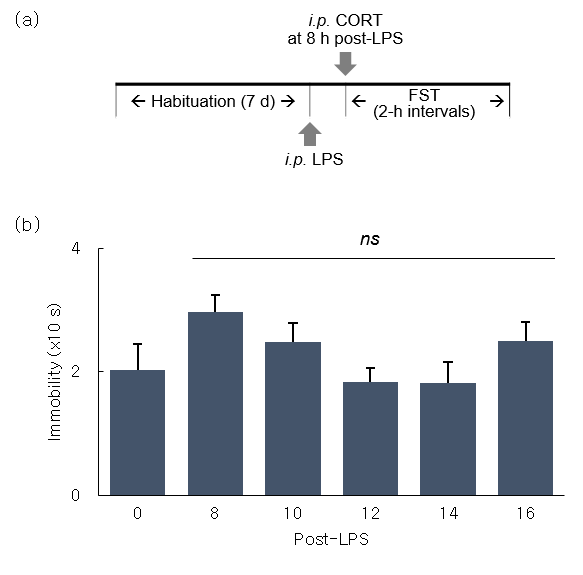


**Supplemental Figure 6. Effect of CORT pre-treatment on the late depressive-like behavior.** (a) Schedule of experimental procedures. (b) The duration of immobility time in the FST. Six group mice were treated *i.p.* with 10 mg/kg CORT [n = 7 /group for FST, excepts 0 h group (n =6)], and then the immobility was measured every 2 h for a period of 10 h, beginning at 8 h post-LPS. CORT pre-treatment abrogated the depressive-like behavior induced by LPS. Data represent means ± SEM. *ns* = non-significant by Student’s t-test.


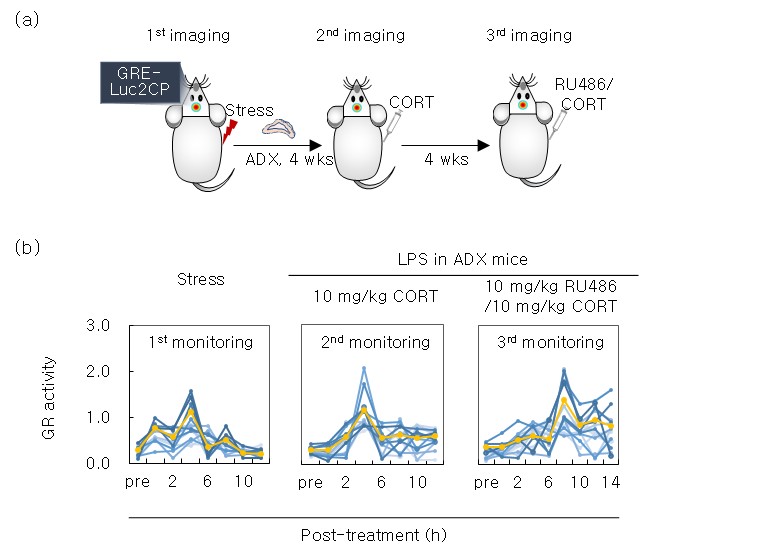


**Supplementary Figure 7.** **Temporal effect of systemic RU486 pulse on the GR activity in ADX mice.** (a) Schematic illustrating repeated analysis with single-monitoring in the IL-PFC (n = 13). (b) Profiles of GR activity of the same ADX mice with stimulators including 2 h of IMO stress, 10 mg/kg CORT, and 10 mg/kg RU486 + 10 mg/kg CORT. RU486 was administered 1 h before CORT treatment in the 3^rd^ monitoring. Orange lines indicate the average activity signal at each time point. ADX, Bilateral adrenalectomy; CORT, corticosterone.


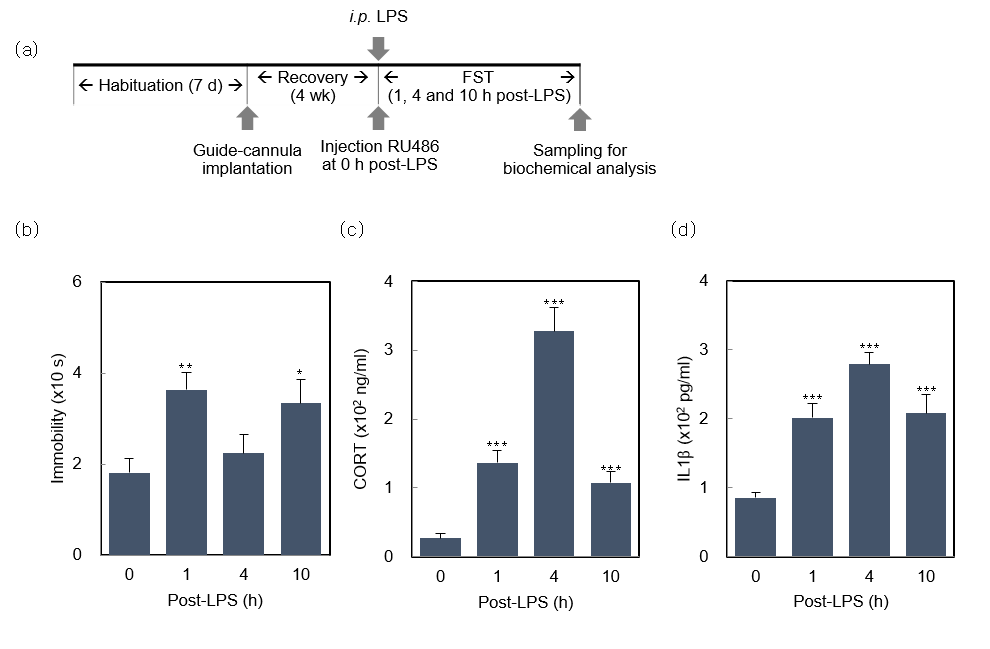


**Supplementary Figure 8. Effect of direct microinjection of RU486 into the IL-PFC.** (a) Schedule of experimental procedures. (b) The duration of immobility time in the FST. (c) Plasma CORT. (d) Plasma IL1β. Microinjection of 10 ng RU486 (500 ng/kg) was injected into the right IL-PFC, before *i.p.* injection of 1 mg/kg LPS. Data are represented as the mean ± standard error of measure (n = 5/each time point). Statistical significance was determined by Student’s t-test (**P* < 0.05, ***P* < 0.01, ****P* < 0.001 *vs* control).


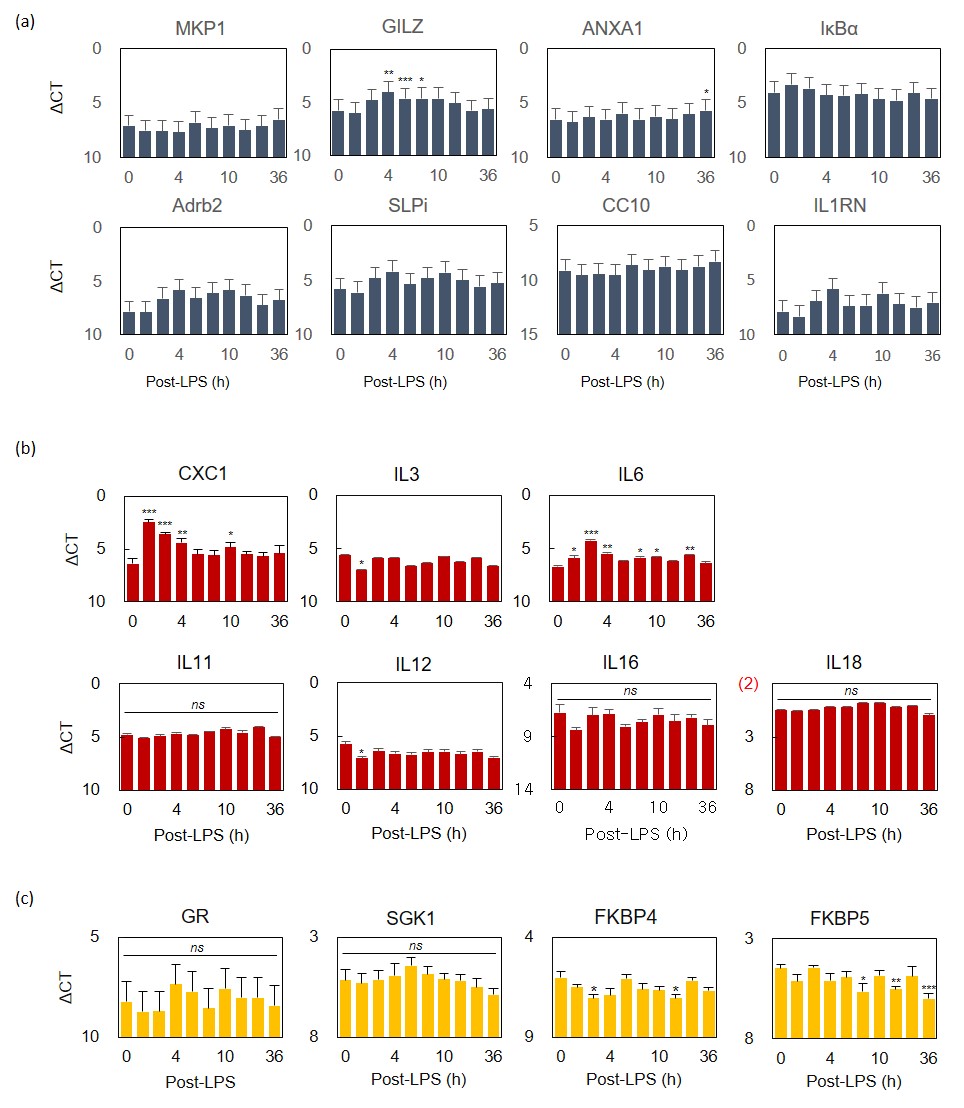


**Supplementary Figure 9. Temporal changes in mRNA expression by qPCR.** (a) 8 GR-specific genes known to inhibit NFκB signaling and (b) 7 NFκB-response genes which were used in the multiplex connectivity analysis. (c) GR gene and GR activity modulated genes. Statistical significance was determined by Student’s t-test (**P* < 0.05, ***P* < 0.01. and ****P* < 0.001 *vs* control).


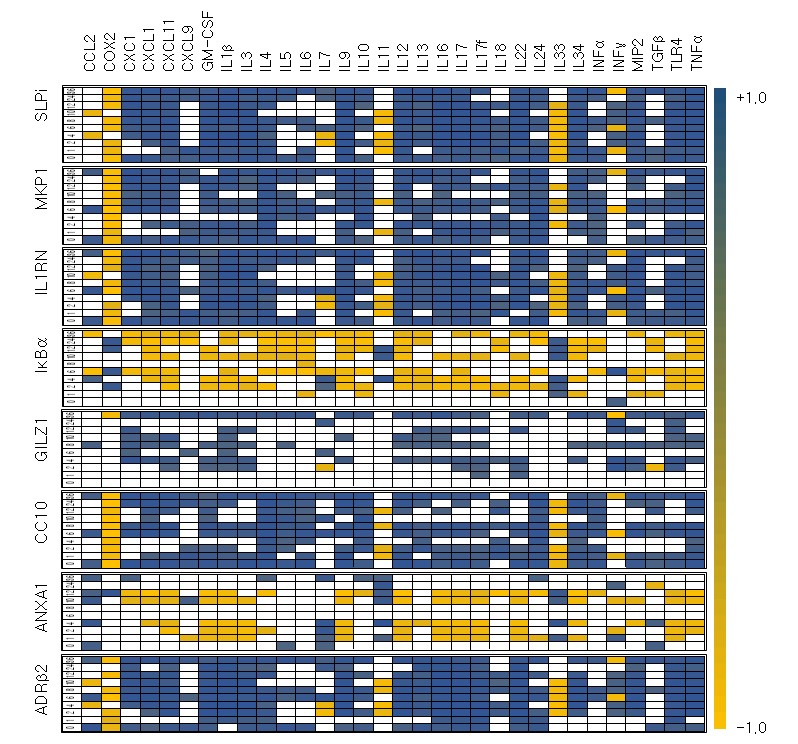


**Supplementary Figure 10.** **Time-lapse heat map for NFκB-GR correlation coefficient.** Correlation analysis of NFκB-GR transcripts was performed for 8 GR-specific genes (Adrβ2, ANXA1, CC10, GILZ, IκBα, IL1RN, MKP1, and SLPi). This demonstrated that ANXA1 and IκBα had negative-dominant correlations coefficient to the cytokine genes, but the others were predominately positively correlated. These results enable us to understand the GR transcriptional inhibition effect on each of the 32 cytokine genes. Significant correlation coefficients (*P* < 0.05) from GraphPad Prism (GraphPad Prism software v7.04 Inc.) were used in this analysis.


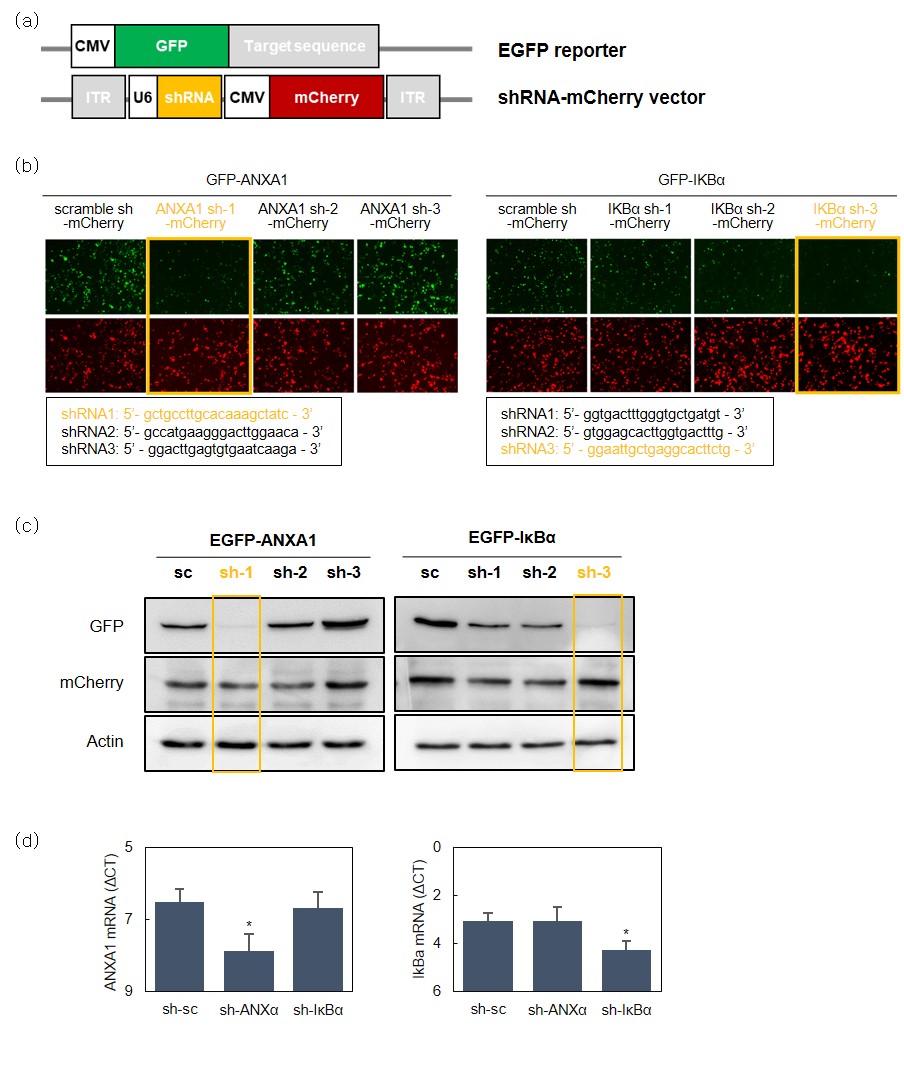


**Supplementary Figure 11. Screening of candidate shRNAs for ANXA1 and IκBα silencing in vitro.** (a) Schematic of GFP reporter and shRNA-mCherry vectors. (b) In vitro fluorescence images for shRNA target gene expressions. Green; target gene expression, red; shRNA expression. HEK293 cells were co-transfected with the fusion reporter construct expressing EGFP and shRNA-mCherry vectors targeting ANXA1 or IκBα. (c) In vitro confirmation of gene knockdown by Western blot assay. The orange box represents the candidates for in vivo knockdown. (d) *In vivo* validation with qPCR analysis of the ANXA1 or IκBα knockdown in the IL-PFC. Expression changes are presented relative to sh-scramble group (n = 11 for the in vivo validation). Statistical significance was determined by Student’s t-test (**P* < 0.05 *vs* sh-sc).


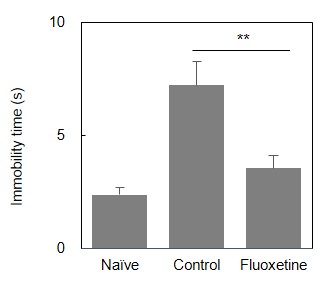


**Supplementary Figure 12. Pharmacological validation of the FST in the LPS-induced depression ICR mouse model.** ICR mice were divided into three groups as follows: naive mice, control (1 mg/kg LPS) and fluoxetine group (10 mg/kg fluoxetine + 1 mg/kg LPS). Depressive-like behavior was analyzed by observations at 12 h following systemic LPS administration. Fluoxetine treatment significantly reversed LPS-induced depression-like behavior, suggesting that the ICR mouse model is sufficient for the behavioral testing studies. Data are shown as mean ± standard error of the mean (SEM). Data are represented as the mean ± standard error of measure (n = 10 for naïve and n = 9 for control and fluoxetine group). Statistical significance was determined by Student’s t-test (***P* < 0.01).
